# Detection and Analysis of the Oral Flora in Patients with Recurrent Aphthous Stomatitis

**DOI:** 10.1155/2022/1705193

**Published:** 2022-10-13

**Authors:** Ruolin Cai, Wei Bi, Youcheng Yu

**Affiliations:** ^1^Department of Stomatology, Zhongshan Hospital (Xiamen), Fudan University, Xiamen 361015, China; ^2^Department of Stomatology, Zhongshan Hospital, Fudan University, Shanghai 200032, China

## Abstract

The present study detected differences in the oral mucosal flora in healthy people and patients diagnosed with recurrent aphthous stomatitis (RAS) using the 16S ribosomal RNA high-throughput sequencing (rRNA-seq). All samples were collected from the lower lip mucosa of 100 healthy individuals and 100 patients with RAS. After the extraction, DNA was subjected to PCR amplification for the bacterial 16S rRNA gene, before subjecting to high-throughput sequencing, and matched to a database. Most bacterial species and most unique bacteria were from the healthy control group, and the amount of operational taxonomic units (OTUs) calculated was similar in the ulcer and nonulcer sites. *Firmicutes*, *Proteobacteria,* and *Bacteroidetes* were most abundant in the healthy group and in ulcer, nonulcer, and healed ulcer sites at the phylum level. Meanwhile, the number of *Prevotella* was significantly elevated in ulcer sites (*P* < 0.05). Healthy people had more species of bacteria inhabiting their oral mucosa than did RAS patients, and patients with ulcers had the lowest abundance of bacterial species. We suggest that the number of *Prevotella* is associated with RAS.

## 1. Introduction

Recurrent aphthous stomatitis (RAS) is a chronic inflammatory common ulcerative disease in the oral mucosa with an incidence ratios of ten to twenty percent [1]. RAS was classified into three categories according to different clinical presentations: herpetiform (multiple small pinpoint ulcers), major (ulcers >10 mm in diameter), and minor (ulcers ≦10 mm in diameter) [2]. The general clinical characteristics include an oval/round shallow ulcer, with a gray-white pseudomembrane on the surface and a thin erythematous halo envelope, with obvious pain. The pathogenesis is primarily divided into the attack period (prodromal period/ulcer period), healing period, and intermittent period, with different durations. RAS is generally self-limiting. The predilection sites of RAS are the lips, tongue, and gingiva. Noticeably, RAS is extremely painful, and the healing time is longer than traumatic ulcers of the same size [3]. RAS affects the normal functions of the oral cavity, such as eating and speaking, as well as teeth brushing.

The causes of RAS are not clear, and there are obvious individual differences. Recent studies suggested that the occurrence of RAS was due to local inflammation caused by an imbalance in the oral mucosal flora [2]. Aas et al. found that the predominant bacterial flora in the healthy oral cavity was highly variable between people and was site-specific [1]. Seoudi et al. found that colonization of *Rothia dentocariosa* was increased at nonulcer sites in RAS patients, and the oral mucosa of healthy controls (HCs) had higher abundances of *Neisseria* and *Veillonella* than the patient groups [4].

However, study results are regionally variable. For example, Hijazi showed that oral Streptococcaceae species were predominant in HCs compared to the ulcerated sites of RAS patients but not in HCs compared with uninfected sites in RAS patients in the UK [5]. In contrast, Bankvall showed that the noninflamed buccal mucosa' microbiota varied between HCs and patients in Gothenburg, Sweden [6]. Yun-Ji found that increased *Acinetobacter johnsonii* and decreased *Streptococcus salivarius* in the mucosa were related with RAS risk in Koreans [7]. Some scholars believe that *Helicobacter pylori* infection is linked with an increased risk of RAS [8, 9], but others believe that *Helicobacter pylori* is not directly related to RAS [10].

Relevant literature suggests that oral mucosal flora differs between healthy people and patients with RAS, but the detection results in patients with RAS vary between studies, which may be due to the use of different populations and detection methods.

Many methods for bacterial species analysis are emerging. High-throughput sequencing is a relatively advanced and accurate method that is becoming increasingly mature, and it is able to simultaneously sequence massive DNA molecules up to millions of molecules. High-throughput sequencing method enabled us to perform a detailed and comprehensive analysis of not only the transcriptome but also the genome of a species. Therefore, this study utilized high-throughput sequencing method to compare variations in oral mucosal flora between patients with RAS and healthy group.

## 2. Materials and Methods

The ethics committee of Zhongshan Hospital, Fudan University, Shanghai, China approved this study. All parents in this research were noted of the purpose and signed informed consent in written. The oral medicine specialist diagnosis is minor aphthous ulcer.

### 2.1. Subject Selection

This experiment recruited patients at Zhongshan Hospital, Fudan University, Shanghai, China from September 2018 to August 2019. All volunteers underwent strict physical, laboratory, and oral examinations.

The inclusion criteria as follows:

1. Healthy volunteers with confirmed results from physical, laboratory, and oral examinations.
2. Confirmed results from systemic and laboratory examinations (oral disease was diagnosed as RAS from the patient's history of treatment and clinical examination).
3. Lived in Shanghai for more than five years.

The exclusion criteria were as follows:

1. Smoking or drinking history.
2. Pregnancy or lactation.
3. The presence of chronic systemic diseases.
4. Abnormal red blood cell, vitamin B12, vitamin D, folate, ferritin, iron ion, or zinc ion indexes.
5. Any antibiotics taken in the preceding 3 months.
6. Any kind of treatment for RAS in the preceding six months.
7. The use of any medication.
8. The presence of other mucosal diseases.
9. Dental caries.

### 2.2. If There Is a Periodontal Disease

1. A high-sugar diet.
2. Go to bed after 23 : 00.

All participants were banned from using antibacterial mouthwashes, and they were asked to brush their teeth twice daily for two weeks before sampling. At the time of sampling, the participant's ulcer is minor ulcer (diameter: 5∼10 mm).

### 2.3. Sample Collection

Time point of all the sample collection is in the morning. The same operator used a sterile throat swab to repeatedly scrape the lower lip mucosa 5 times, while ensuring that the throat swab did not touch the teeth and other parts during the sampling process. Direct swabs of healthy sites on the lower lip mucosa in RAS patients and HCs were collected. Swab samples were also collected from ulcers on the lower lip mucosa in RAS patients, and these sites were sampled again when the ulcer healed. The samples were stored in a −20°C freezer. The swabs did not touch the teeth at the time of sampling.

### 2.4. Extraction and Amplification of DNA

Total DNA was extracted under the instructions of the EZNA® Soil Kit (Omega Bio-Tek, Norcross, GA, US). DNA purity and concentration were assessed by a NanoDrop2000, and the quality of the extracted DNA was examined using DNA electrophoresis. Using the primers 806R (5′-GGACTACHVGGGTWTCTAAT-3′) and 338F (5′-ACTCCTACGGGAGGCAGCAG-3′), the polymerase chain reaction amplification of the V3-V4 variable region was performed using an ABI GeneAmp® 9700 and the program 95°C predenaturation for 180s was carried out, and then 27 cycles of 95°C denaturation for 30 s, annealing for 30s at 55°C, and extension for 30s at 72°C, with an extension for 600s at 72°C were carried out. Followed by the 20-*µ*l volume amplification system includes 10 ng DNA template, 2 *µ*l 2.5 mM dNTPs, 4 *µ*l of 5x FastPfu buffer, 0.4 *µ*l FastPfu polymerase, and 0.8 *µ*l primer (5 *µ*M).

### 2.5. Illumina MiSeq Sequencing

The PCR products were enriched by electrophoresis of 2% agarose gel, and an AxyPrep DNA Gel Extraction Kit (Axygen Biosciences, Union City, CA, USA) was applied to purify the PCR product, followed by the elution with Tris-HCl. After detection of DNA using 2% agarose electrophoresis, QuantiFluor™-ST (Promega, USA) was utilized for quantification of PCR product. The amplified fragments were applied to build a library of PE 2∗300 according to the standard procedures provided by the Illumina MiSeq platform.

The following steps were used to construct the library: (1) connect the “Y”-shaped adapter; (2) screen and remove the self-linking fragments of the adapter using magnetic beads; (3) enrich the library template using PCR amplification; and (4) produce single-stranded DNA fragments through denature using sodium hydroxide.

Sequencing was completed through Illumina's MiSeq PE300 platform (Shanghai Meiji Biomedical Technology Co, Ltd), with the original data transmitted to the database of NCBI.

### 2.6. Processing of Sequencing Data

This research applied Trimmomatic software to control the original sequencing, and the software FLASH was utilized for splicing, as follows:

1. Set a window of 50 bp. If the average value of quality was below 20 in the window, all the later sequences in the window were removed from the front, and the sequences were removed after quality control if its length did not reach 50 bp.
2. The sequences were joined at both ends according to the overlapping bases. During splicing, the max mismatching ratio was 0.2 between overlaps, with its length ≥10 bp. Sequences were either joined or removed.
3. The sequence was split into each sample according to the primer and the barcode at both or either end of the sequence. The barcodes were exactly matched. The primer allowed mismatches of 2-base, while sequences were removed if ambiguous bases exist.

The software UPARSE was utilized for performing operational taxonomic unit (out) clustering of sequences at a sequence similarity of 97%, and a single chimera or a sequence was eliminated during the process of clustering. The RDP classifier was used to annotate and classify sequence and perform comparisons with the comparison threshold set to 70% in the Silva database (SSU123).

## 3. Results

The two hundreds enrolled subjects included 100 RAS patients and 100 HCs. Half of the RAS group was male, and the range of their age is 37 ± 9.6 years. Half of the HC group was male with the age range of 43 ± 7.9 years (Table 1).

**Figure 1:**
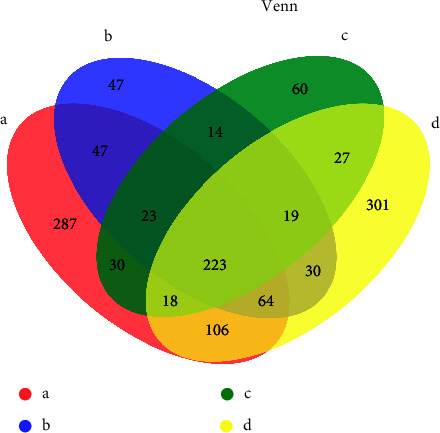
Comparison of oral microbial communities between groups using Venn diagrams. (a) Healthy group; (b) ulcer site; (c) nonulcer site; (d) healed ulcer site.

A Venn diagram in Figure 1 illustrated that 223 OTUs appeared in all the four groups. There were 357 OTUs in the healthy group (a) highly similar to the ulcer site (b) in the RAS group during the ulceration stage; 294 OTUs in the healthy group (a) and the nonulcer site (c) were highly similar; 411 OTUs were highly similar in the healthy group (a) and the healed ulcer site (d); 279 OTUs were highly similar between the ulcer site (b) and the nonulcer site (c) in the RAS group during ulceration; 336 OTUs were highly similar between the ulcer site (b) and the healed ulcer site (d); 287 OTUs were highly similar between the nonulcer site (c) and the healed site (d).

The healthy group (a) had the highest number of OTUs, with 798; the ulcer site had the least number of OTUs, with 467. Meanwhile, the healthy group had the highest unique OTUs, with 287. The account of OTUs was similar between the healthy group (a) and the healed ulcer sites (d), and the numbers of OTUs in these two groups were highest.

**Figure 2:**
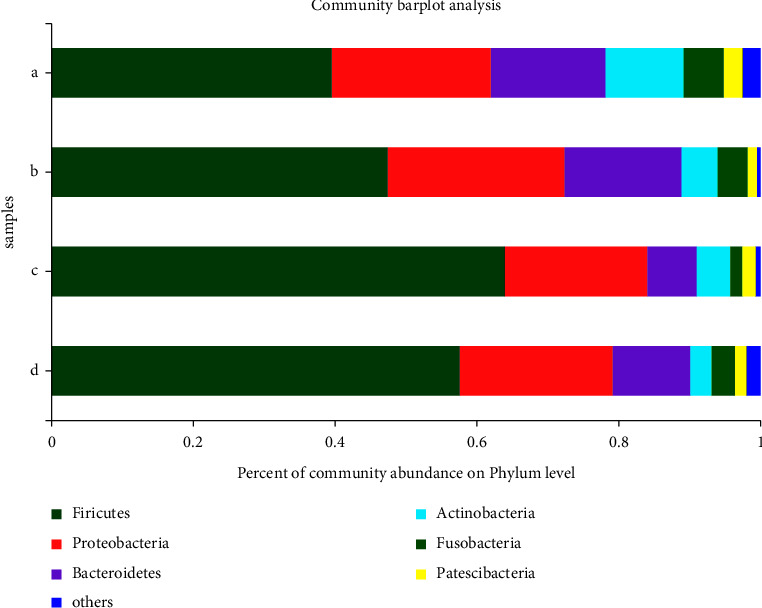
Relative abundance of species in the oral microbial community at the phylum level of each group. (a) Healthy group; (b) ulcer site; (c) nonulcer site; (d) healed ulcer site.

Phylum-level comparisons between flora composition and relative richness showed that *Firmicutes*, *Proteobacteria*, *Actinobacteria*, *Bacteroidetes*, *Fusobacteria*, and *Patescibacteria* were the first six dominant bacterial phyla (Figure 2). The most abundant species was *Firmicutes* in all four groups. *Patescibacteria*, *Actinobacteria,* and *Fusobacteria* were more abundant in the healthy group. Much more *Actinobacteria* and *Fusobacteria* were present in the healthy group. *Firmicutes* was much more abundant than the other phyla at nonulcer sites, and *Proteobacteria* was much more abundant than the other phyla at ulcerated sites.

**Figure 3:**
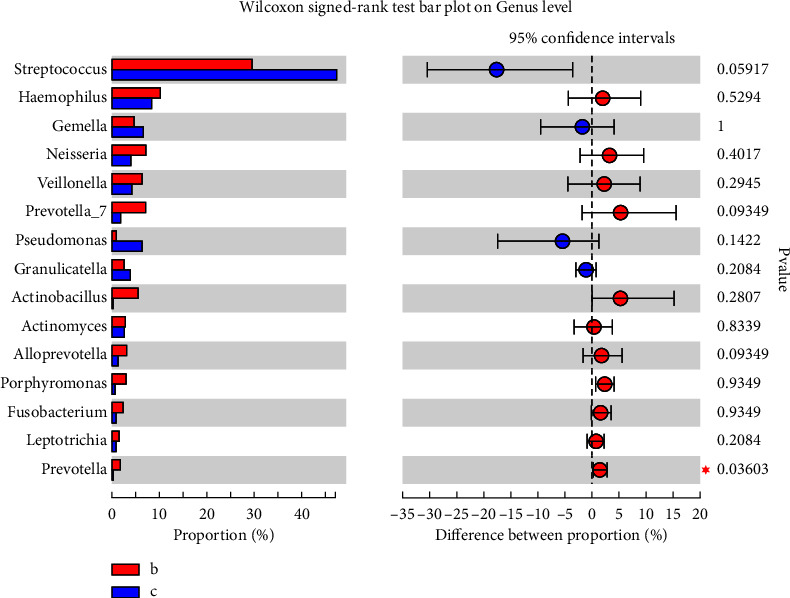
Comparison of the oral microbiota between ulcer and nonulcer sites at the genus level. (b) Ulcer site; (c) nonulcer site. ^*∗*^*p* < 0.05.

**Figure 4:**
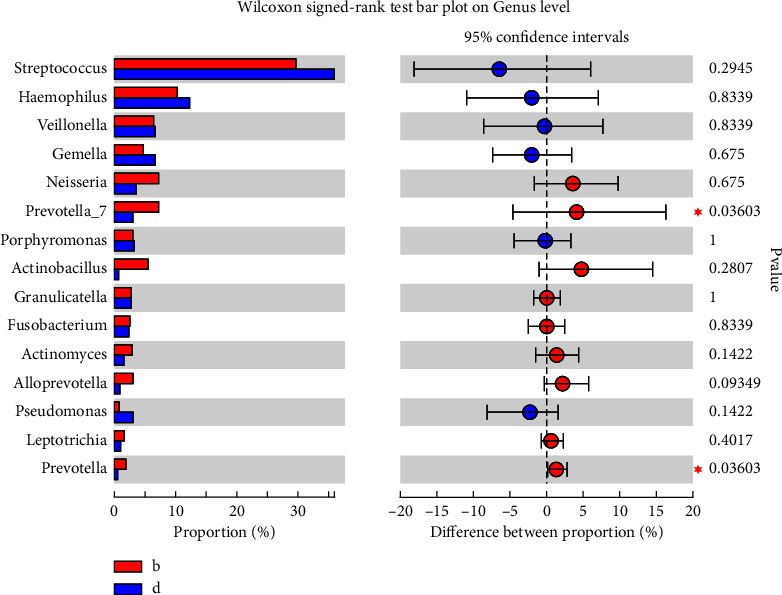
Comparison at the genus level of the oral microbiota between ulcer and healed ulcer sites. (b) Ulcer site; (d) healed ulcer site. ^*∗*^*p* < 0.05.

Significant differences for some bacteria were revealed by genus-level diversity comparisons of high-throughput sequencing findings. The abundance of *Prevotella* (*P* < 0.05) at the ulcer sites in RAS patients was increased (Figure 3; Figure 4). Samples from the ulcer sites were relatively independent from the other samples.

## 4. Discussion

The pathogenesis of RAS is not clear. Factors affecting RAS include genetic factors, immunological factors, bacterial factors, dietary habits, hormonal disorders, a lack of micronutrients, mechanical trauma, mental factors, systemic diseases, and oxidative stress. Some studies noted that a lack of micronutrients (e.g., vitamins, serum iron, ferritin, and folic acid) may affect the occurrence and development of RAS, but this result is controversial [11–14]. Some studies showed that vitamin B12 treatment was effective in patients suffering from RAS [13, 15]. A Turkish study on serum zinc and vitamin D reported an association between zinc deficiency and RAS [16]. Another study reported insufficient levels of vitamin D in patients with RAS comparing to HCs [17–20]. Du et al. found that frequent consumption of carbonated beverages and frequent thirst increased the possibility of RAS, and the consumption of nuts provided protection [21].

As for mental factors, studies have shown that the frequency of staying up late and the cumulative duration of staying up late will affect the severity of RAS attacks. At the same time, the length of sleep time may affect mental state and easily lead to depression and anxiety. These negative emotions will aggravate RAS and the frequency of attacks and the severity of ulcers [13, 23–26].

At the same time, taking drugs such as glucocorticoids, thalidomide, and other drugs [27] and suffering from certain autoimmune diseases such as Crohn's disease and PFAPA (periodic fever, aphthous stomatitis, pharyngitis, and adenitis) syndrome also affect RAS [28].

The present study detected folate, ferritin, vitamin B12, vitamin D, iron ions, and zinc ions in all participants. The daily drinking frequency and amount were normal in the RAS group, and they reported no frequent consumption of carbonated drinks, no consumption of nuts, and a light diet on weekdays.

According to the existing articles on RAS flora research, it can be found that the methods of flora detection can be roughly divided into several categories: microscopy, T-RFLP, HOMIM analysis, MALDI-TOF-MS, and high-throughput sequencing. The T-RFLP method is to design universal primers based on the conserved regions of bacteria, in which the 5′ end of one primer is labeled with a fluorescent substance. Extract the DNA of the sample to be analyzed and perform PCR amplification to obtain a PCR product with a fluorescent label, which is then digested with the corresponding restriction enzymes. The nucleotide sequences are also different, resulting in different positions of restriction sites, so the lengths of the restriction fragments produced are also different. Then, the sequencer is used for determination, and the fluorescent label at the end of the fragment will be detected, resulting in different peaks, each of which represent at least one bacterium or several closely related bacteria. The HOMIM analysis method is to directly extract the total DNA from the sample and directly perform detection and analysis through the chip. MALDI-TOF-MS isolates and cultivates the bacterial flora of the sample then extracts the polypeptides of the bacteria for detection, and compares them according to the information in the database to obtain the type of bacteria. The principle of the high-throughput sequencing method has been described in “Materials and methods”. High-throughput sequencing is a relatively advanced and accurate method, and the sequencing technology is becoming more and more mature. It can sequence hundreds of thousands or even millions of DNA strands at the same time. Therefore, the detection method of this experiment adopts high-throughput sequencing technology to ensure the accuracy of the experimental data.

In the present study, HCs possess the largest amount of oral mucosa flora species. In contrast, the number of oral flora species in RAS patients decreased by different degrees at different stages during the disease course. During the ulcer stage, both ulcerated (467 OTUs) and healthy sites (414 OTUs) had the fewest bacterial flora species. The number of oral flora increased during the intermittent stage (788 OTUs). At the phylum level, six dominant species of oral mucosa were highly similar between the group of RAS patients and the group of healthy control. However, the dominant species levels differed between the four sites. The flora at the ulcer site in RAS patients was different from the other sites. Previous studies inquired into the composition of the oral bacterial community within healthy controls, while the RAS patients may have suffered from background noise due to the individual differences [22]. Therefore, the present study compared the oral microbiota within the same individual. We found that the increase in *Prevotella* abundance within communities of bacteria was strongly associated with RAS (*P* < 0.05). *Prevotella* belongs to *Bacteroidetes*, which is a Gram-negative anaerobe. The increase in the number of *Prevotella* during the onset of RAS may disrupt the local microecological balance.

## 5. Conclusion

The results of this experiment showed differences in the oral mucosal flora between the healthy control and RAS groups and suggested that the increased presence in *Prevotella* was correlated with RAS.

## Figures and Tables

**Table 1:** Distribution of research subjects according to age and gender.

		HC group	RAS group
Age (years)		43 ± 7.9	37 ± 9.6
Gender	Male	50	50
	Female	50	50

## Data Availability

The data used for the study are included within the article.
